# GenGIS 2: Geospatial Analysis of Traditional and Genetic Biodiversity, with New Gradient Algorithms and an Extensible Plugin Framework

**DOI:** 10.1371/journal.pone.0069885

**Published:** 2013-07-29

**Authors:** Donovan H. Parks, Timothy Mankowski, Somayyeh Zangooei, Michael S. Porter, David G. Armanini, Donald J. Baird, Morgan G. I. Langille, Robert G. Beiko

**Affiliations:** 1 Faculty of Computer Science, Dalhousie University, Halifax, Nova Scotia, Canada; 2 Australian Centre for Ecogenomics, School of Chemistry and Molecular Biosciences, The University of Queensland, Brisbane, Queensland, Australia; 3 Prothea srl, Milan, Italy; 4 Environment Canada @ Canadian Rivers Institute, University of New Brunswick, Fredericton, Canada; Argonne National Laboratory, United States of America

## Abstract

GenGIS is free and open source software designed to integrate biodiversity data with a digital map and information about geography and habitat. While originally developed with microbial community analyses and phylogeography in mind, GenGIS has been applied to a wide range of datasets. A key feature of GenGIS is the ability to test geographic axes that can correspond to routes of migration or gradients that influence community similarity. Here we introduce GenGIS version 2, which extends the linear gradient tests introduced in the first version to allow comprehensive testing of all possible linear geographic axes. GenGIS v2 also includes a new plugin framework that supports the development and use of graphically driven analysis packages: initial plugins include implementations of linear regression and the Mantel test, calculations of alpha-diversity (e.g., Shannon Index) for all samples, and geographic visualizations of dissimilarity matrices. We have also implemented a recently published method for biomonitoring reference condition analysis (RCA), which compares observed species richness and diversity to predicted values to determine whether a given site has been impacted. The newest version of GenGIS supports vector data in addition to raster files. We demonstrate the new features of GenGIS by performing a full gradient analysis of an Australian kangaroo apple data set, by using plugins and embedded statistical commands to analyze human microbiome sample data, and by applying RCA to a set of samples from Atlantic Canada. GenGIS release versions, tutorials and documentation are freely available at http://kiwi.cs.dal.ca/GenGIS, and source code is available at https://github.com/beiko-lab/gengis.

## Introduction

Phylogeography aims to relate evolutionary processes to spatial, temporal and environmental factors in order to understand past and present biodiversity [1]–[3]. Purpose-built software tools allow the geographic analysis of biodiversity by incorporating phylogenetic information in different ways. Several programs provide visualizations of 2D or 3D geophylogenies, along with functionality to explore the temporal structure of this data [3], [4] or the geographic uncertainty associated with Bayesian techniques for phylogeographic inference [5]. Other programs have focused on displaying the spatial distribution and diversity of taxa using pie charts or geographic heat maps, and incorporate phylogenetic information by focusing on specific lineages or assigning molecular sequences to haplotypes [6]–[8]. We developed GenGIS [9], [10] as a tool to merge geographic, ecological and phylogenetic biodiversity data in a single interactive visualization and analysis environment which supports a wider range of visualization and analysis options than existing software packages. Since its release, GenGIS has been used to investigate the phylogeography of viruses [11], [12], bacteria [13], plants [14], [15], animals [16]–[18], humans [19], and language families [20].

Within GenGIS, potential migration routes or the influence of geography on community similarity can be explored by proposing linear or non-linear geographic axes, and visualizing the goodness of fit between a tree topology and the ordering of sample sites along the specified axis [21]. The first version of GenGIS required the user to draw geographic axes by hand, allowing the testing of specific hypotheses but making it difficult to explicitly test all possible axes. The authors of [15] noted that “GenGIS does not allow broad testing of encoded hypotheses with automatic polyline enumeration” and manually tested a subset of all possible axes for a set of georeferenced kangaroo apple samples. We have developed version 2 of GenGIS, a major new release with improved stability and functionality on both the Windows and Macintosh platforms. GenGIS v2 now includes an automated test of *all* possible linear geographic axes and a new plugin architecture that allows the scripting and execution of custom data analyses. Included with GenGIS are plugins implementing widely used statistical approaches such as the Mantel test, several alpha diversity measures, and geographic visualizations of dissimilarity matrices. We have also developed a plugin that implements the Atlantic Canada river reference model [22], which demonstrates the use of GenGIS in ecosystem biomonitoring, an area currently being transformed by genomics approaches [23]–[24].

Here we illustrate the new features of GenGIS using three datasets. We first demonstrate the application of new gradient methods to the kangaroo apple dataset of [15] to find the optimal geographical axis and test its significance. The functionality of several plugins is demonstrated using microbial community data from 28 different body sites sampled by [25], revealing alpha-diversity patterns by body site and clustering fecal samples from different individuals. Finally, we demonstrate the application of the Atlantic Canada reference model on benthic macroinvertebrate samples from a series of 16 potentially impaired sites in Nova Scotia.

## Methods

### Data Acquisition and Formats

GenGIS makes use of digital map data, sample site information, sequence data, and one or more phylogenetic trees. Digital map data can be freely obtained from several online sources as described on the GenGIS website (http://kiwi.cs.dal.ca/GenGIS), or using our freely available program, MapMaker. A wide range of raster file formats are supported, including both digital elevation maps for visualizing 3D terrain and georeferenced image files for displaying standard map or satellite imagery. GenGIS also supports vector files and allows these to be overlaid on a raster map. The locations file indicates the geographic coordinates of each sample site, along with other relevant site attributes (e.g., habitat parameters, sampling details). Information about sequences, such as taxonomy or molecular function, collected at each sample site can be specified in an optional sequence file. Tree files are automatically georeferenced by associating leaf node names with the unique identifier used to specify either the sample sites or sequences. Complete descriptions of file formats and example data files are available on the GenGIS website. GenGIS v2 also allows the state of a previous session to be restored from a file.

### Phylogeographic Techniques

**Figure 1:**
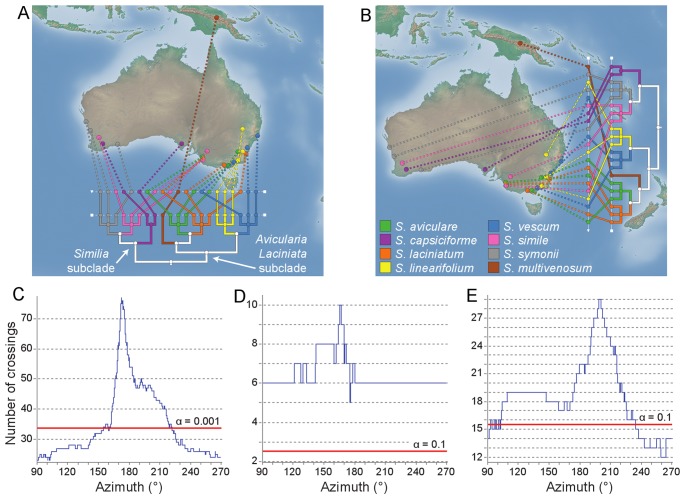
Phylogeography of kangaroo apples. A) A longitudinal gradient resulting in 23 crossings. Each of the eight species within the kangaroo apple phylogeny is assigned a unique color, and the two most substantial subclades are labelled. B) A latitudinal gradient results in 57 crossings. C) Results of a linear axes analysis on the kangaroo apple dataset. The number of crossings is only shown for axes between 90° and 270° as the graph has a period of 180°. Under the null model, only 10 of 10,000 permutations resulted in fewer than 34 crossings which is depicted by the red line (i.e. α = 0.001). D) A linear axes analysis of the *Similia* subclade with the red line set to reflect a conservative critical value of α = 0.1. E) A linear axes analysis of the *Avicularia/Laciniata* subclades (α = 0.1).

A linear axis defines an ordering of sample sites based on their projection onto the specified geographic gradient (Fig. 1a; see also [10], [21] for a detailed description of the algorithm). The fit between a tree and a geographic gradient is determined by finding the ordering of leaf nodes, subject to the constraints of the tree topology, which most closely matches the ordering of sample sites. When these two orderings are shown in parallel, any mismatches between them will cause crossings between the lines connecting leaf nodes to their associated sample sites. The optimal tree layout is the one resulting in the fewest crossings. We previously proposed a branch-and-bound algorithm which allows the globally optimal layout to be found for large, multifurcating trees (∼1000 leaf nodes with an average node degree <8) in interactive time, i.e. <100 ms [21]. A permutation test can be used to assess the expected number of crossings under a null model of no geographic structuring [21]. This test performs 1000 permutations of the tree leaf labels and calculates the optimal layout and number of crossings for each of these permutations to generate a null distribution. The p-value is equal to the proportion of permuted trees that yield fewer or the same number of crossings as the tree with unpermuted labels.

In GenGIS v2, we have developed a method, *linear axes analysis*, for efficiently determining the number of crossings which occur for any linear axis. The proposed method is a plane sweeping algorithm where a line (i.e., the linear axis) is rotated by 180° [26]. A 180° sweep is sufficient as the ordering of sample sites along a gradient at angle θ will be identical to the ordering at θ+180° (i.e., gradients are treated as being undirected). The key insight of the proposed algorithm is observing that the ordering of sample sites along the sweep line changes only when the line between a pair of sites becomes perpendicular to the sweep line. This suggests an 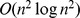
algorithm for determining the number of crossings which occur for any linear axis. For each of the 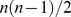 pairs of sample sites, the slope of the line connecting the sites and the site information is stored in an array. This array is then sorted in ascending order of slope values. Starting from a horizontal sweep line where sample sites are ordered by their longitudinal (x-axis) position, the sorted slope array indicates the order in which sample sites must be swapped as the sweep line is rotated. For each permutation of the sample sites, the optimal tree layout is determined and the number of crossings for the current orientation of the sweep line stored (see Fig. 1c for a plot of such data). Pseudocode along with a discussion of degenerate cases (e.g., collinear points) is given in the Supplementary Material.

### Visualization and Data Analysis

The Python console in GenGIS allows users to interact directly with data through the GenGIS application programming interface, and allows analyses to be performed using the scipy (http://www.scipy.org/) and numpy (http://numpy.scipy.org/) libraries. Users can also execute commands in the R statistical programming language (http://www.r-project.org) via the RPy2 library. New in GenGIS version 2 is a framework that allows users to develop plugins that provide a graphical interface for frequently used operations. Plugins can import external files and produce visualizations in both the plugin window itself and the map environment. Default plugins in GenGIS include regression analysis, the Mantel test, alpha-diversity calculation, and visualizations of external matrices indicating pairwise site dissimilarities.

### Reference Condition Analysis

We used the Atlantic Canada reference model [22] to assess whether a range of potentially impacted sites had reduced biodiversity below what would be expected based on a set of reference sites. The reference model was trained using a River Invertebrate Prediction and Classification System (RIVPACS) approach [27], [28]. This approach predicts the expected taxon composition at a site from environmental conditions observed at the site, using prior knowledge of taxonomic occurrence at reference sites with similar habitat properties. For a test site, the comparison between observed composition (O) and expected composition (E) - summarized as an O/E statistic - gives an indication of the divergence of the test site from the predefined reference condition. An O/E value close to or greater than 1.0 suggests that the taxon composition at the test site does not deviate substantially from the expected composition, given the observed habitat properties. Conversely, an O/E value substantially less than 1.0 indicates reduced diversity or richness that may indicate an impacted site. The method is widely used (e.g. USA, Australia) and the approach is incorporated in the European Water Framework Directive.

Four geospatial variables were employed in model building: long-term annual temperature range (°C), % intrusive rocks, % sedimentary rocks, and % sedimentary and volcanic rocks. These variables were employed to derive a Discriminant Function (DF) to support the prediction of the expected taxon composition. By means of comparison with an independent validation dataset, the precision and accuracy level, measured in terms of the root-mean-square error (RMSE) and standard deviation of the O/E ratio, were confirmed to align well with other comparable models. Test data sites were analyzed to compare observed biodiversity with the predictions of the reference model to generate the O/E ratio. The following O/E measures were computed based on the model: (i) Taxon richness O/E; (ii) Shannon diversity O/E; (iii) Pielou’s Evenness O/E; and (iv) Berger–Parker dominance index O/E. To compute the DF-based O/E measure for each index we have extended previous work [22], [29], [30]. Since the focus of the analysis is on impacted sites with O/E <1.0, all O/E ratios greater than 1.0 were constrained to the value 1.0 for the purposes of data visualization.

### Test Datasets

The kangaroo apple analysis was conducted using the phylogeny and sample site information provided by [15]. Map data of Australia was obtained using MapMaker, a companion program to GenGIS that allows custom georeferenced maps to be derived from the digital map data provided by Natural Earth (http://www.naturalearthdata.com/). These data files are provided in the Supplemental Information.

Body site data from [25] were obtained from the DNA Data Bank of Japan (ERA000159). The source data consisted of FASTQ files containing amplicons of variable region 2 (V2) of the 16S ribosomal RNA gene. We used version 7 of the RDP classifier [31] as implemented in mothur 1.16.1 [32] to assign taxonomy to all 16S sequences in this dataset. The resulting taxon counts generated were used to generate visual summaries for each body site. Bray-Curtis distances were calculated for each pair of samples, and the resulting distance matrix subjected to Unweighted Pair Group Method with Arithmetic Mean (UPGMA) clustering in mothur. The background image was modified based on an original image obtained from Wikimedia Commons (http://commons.wikimedia.org/wiki/File:Human_body_silhouette.svg).

RCA was based on benthic macroinvertebrate samples collected between 2002–2011 in the Atlantic Maritime ecozone. Data employed for the example here are described in detail in [22]. Samples were obtained from reference sites included in the calibration dataset used for model construction (n = 128). Reference sites were distributed throughout New Brunswick, Nova Scotia, and Newfoundland. Test sites (n = 16) used for model testing in the present paper were collected in the Upper Mersey area of Nova Scotia. Most macroinvertebrate samples were collected using a standardized traveling kick method, in which the operator disturbs the river substrate to dislodge attached and unattached organisms, which are washed into a triangular net of 400-µm mesh size while zig-zagging upstream. Samples were subsequently sorted in the lab and identified to the taxonomic level of family, to allow the identification of sites deviating from expected assemblage composition. Topographical data were obtained from the Shuttle Radar Topography Mission (SRTM) dataset, via the Oak Ridge National Laboratory Distributed Active Archive Center (ORNL DACC: http://http://daac.ornl.gov/). Overlaid on the topography map was vector data describing rivers in Atlantic Canada, obtained from Geobase (http://www.geobase.ca).

## Results and Discussion

### Biogeography of Kangaroo Apples

The biogeography of kangaroo apples (*Archaesolanum*) in Australia and Papua New Guinea was recently examined by [15]. In their analysis, GenGIS was used to investigate geographic structuring by manually testing a subset of all possible linear axes. Here we demonstrate how the linear axes analysis function in GenGIS v2 allows all possible linear axes to be easily evaluated. Defining a strict west-east axis results in 23 crossings (Fig. 1a), whereas a strict north-south axis produces 57 crossings (Fig. 1b). While this reveals stronger longitudinal than latitudinal structuring, by evaluating all linear axes we can determine the globally optimal axis, and the range of angles over which statistically significant results are obtained. For the kangaroo apple phylogeny, the minimum crossing number of 23 occurs multiple times between 90° and 103°, while the maximum number of crossings (77) occurs around 172° (Fig. 1c). Even with a conservative critical value of α = 0.001, a wide range of axis orientations (90° to ∼150°, and ∼220° to 270°) result in significantly fewer crossings than expected under a random model (Fig. 1c), strongly supporting spatial structuring centered on a longitudinal gradient. A linear axes analysis can also be applied to specific lineages. On the *Similia* lineage (Fig. 1a), no linear axis resulted in fewer crossings than expected under the null model (Fig. 1d). For the *Avicularia/Laciniata* lineage marginally significant (*p*-value <0.1) results were obtained for linear axes slightly south of due east and between ∼230° to 270° (Fig. 1e). The absence of notable longitudinal structuring within either subclade suggests that the strong longitudinal structuring found for the full phylogeny is primarily due to species within the *Similia* subclade being to the west of those within the *Avicularia/Laciniata* subclade.

### Statistical Analysis of Human-Associated Microbes

**Figure 2:**
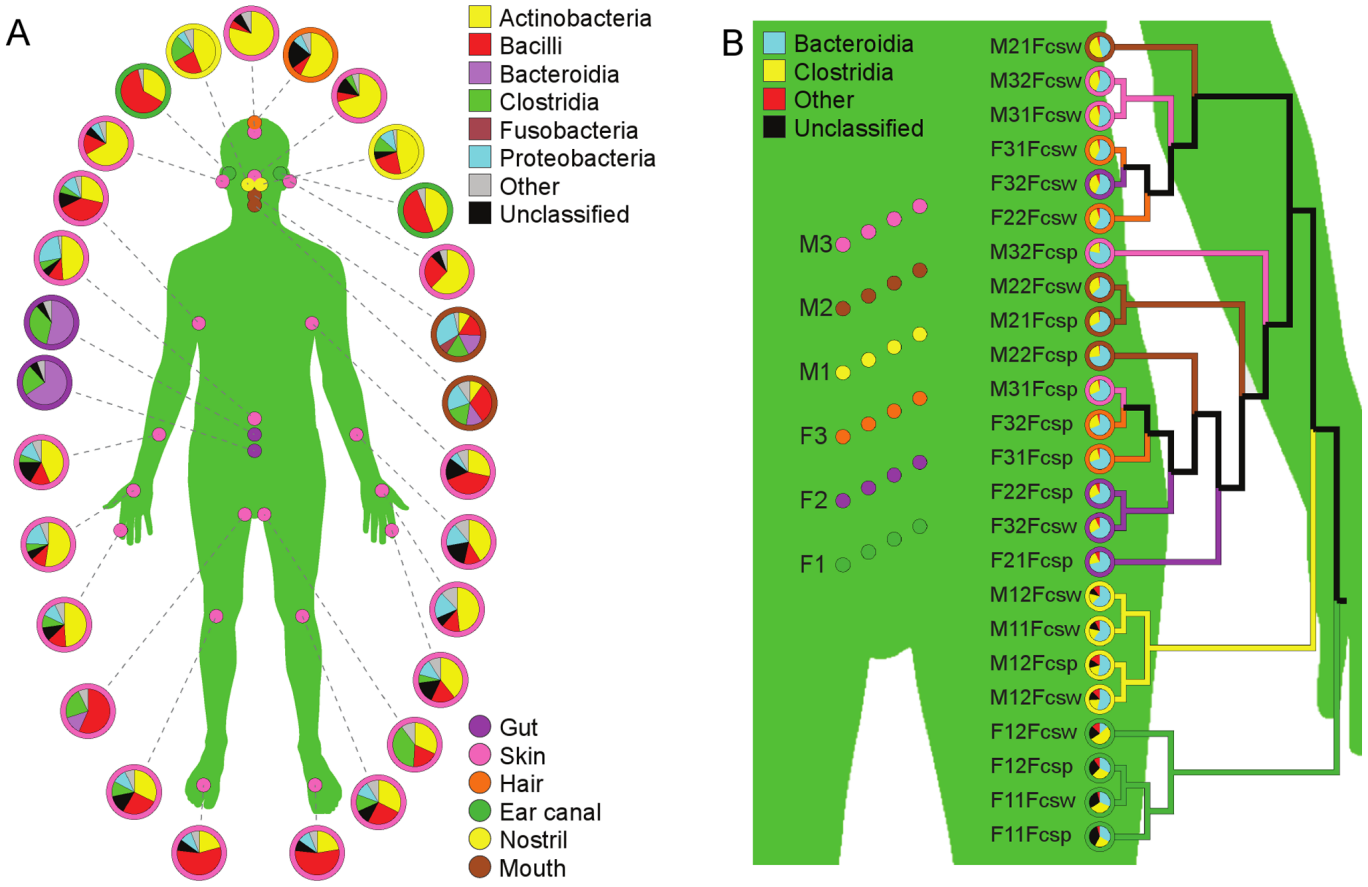
Biogeography of human-associated bacterial communities. A) Summary of taxonomic distribution across 28 different body sites. B) Detailed view of gut microbiota for six individuals (M1, M2, M3 = male; F1, F2, F3 = female). The third digit of each sample indicates time point 1 or 2 for a given individual, while the four-letter identifier indicates the sampling type (Fcsp = swab directly from stool sample, Fcsw = swab of toilet paper). The four samples for each individual are colored identically on the human body map. The UPGMA tree at right indicates the relative similarity of different samples; edges whose children all originate from a single individual are assigned the appropriate color, while black branches cover samples from more than one individual.

**Figure 3:**
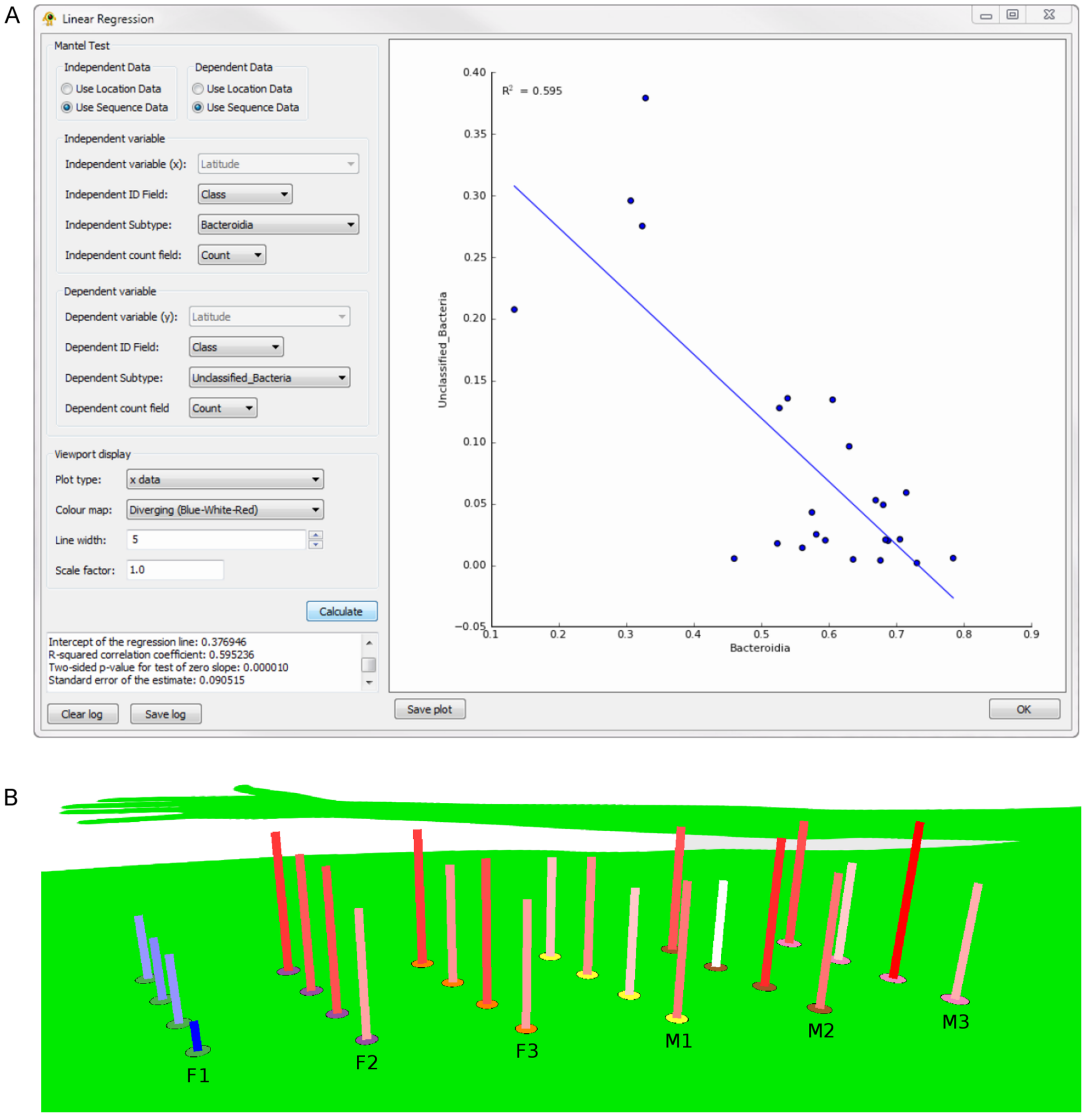
Linear regression analysis of class frequencies in fecal samples from six individuals. A) Linear regression plugin in GenGIS, showing data selection panels in the upper left, quantitative results display (e.g., p-value) in the lower left, and scatterplot on the right. B) Geographic visualization of Bacteroidia frequencies as bar charts with a color gradient (blue = low frequency, white = intermediate frequency, red = high frequency). The arrangement of samples is identical to that in Figure 2b.

The new plugin architecture of GenGIS allows straightforward statistical analysis of biodiversity datasets. The authors of [25] profiled the microbial communities from nine individuals at 28 body sites and several time points using 16S rDNA genes as taxonomic markers, and concluded that community structure for a given individual at any site was relatively stable, although variation was seen among individuals. As reported by the original authors, taxonomic composition varies considerably by body site, with Actinobacteria and Bacilli predominant in many sites, other groups such as Proteobacteria found in some locations, but with gut (fecal) samples dominated by Bacteroidia and Clostridia (Fig. 2a). The dataset we used focused on the gut samples of six individuals, collected in two different ways (from toilet paper or directly from feces) and at two different time points: each individual was therefore associated with a total of four samples. UPGMA clustering of these communities based on differences in class abundance yielded perfectly clustered samples for two individuals (M1 and F1) with relatively high proportions of sequences assigned the label “Unclassified Bacteria” by the RDP classifier, but intermingled samples from the other four individuals based on the relative abundance of Clostridia versus Bacteroidia (Fig. 2b). We then used the linear regression plugin (Fig. 3a) to examine the relationship between different classes: a regression of “Unclassified Bacteria” versus Bacteroidia produced a statistically significant (p = 0.00001) negative association between the two groups (Fig. 3b). This suggests that sequences labeled as “Unclassified Bacteria” may originate from phylum Bacteroidetes, but lack sufficiently similar representatives in the RDP database to allow for precise classification. The regression plugin also generates a geographic bar graph visualization in the main GenGIS window, which shows that individual F1 has the four lowest sample frequencies of Bacteroidia (Fig. 3c).

**Figure 4:**
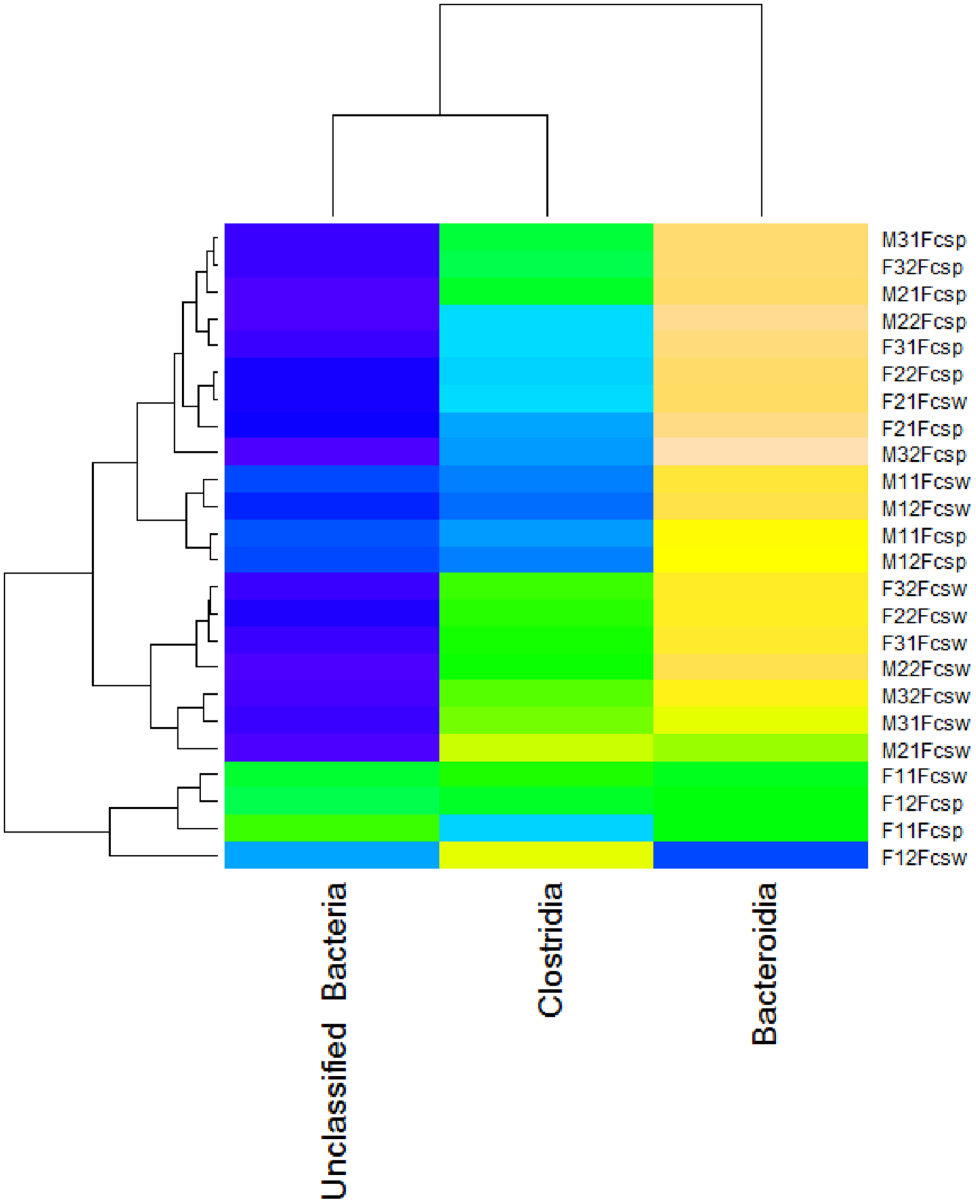
Heatmap of frequencies of three taxonomic groups (Bacteroidia, Clostridia, and “Unclassified Bacteria”) from 24 fecal samples. Dark colors correspond to low frequencies, while yellow, tan and pink indicate high frequencies. Hierarchical clustering of samples and taxonomic groups are shown along both dimensions of the heatmap. Sample labels are explained in the legend of Figure 2b.

Heat maps offer an alternative way to visualize the relative abundance of taxa in different samples, and the “heatmap” function of the R statistical package generates two-dimensional heatmaps with clustering of rows and columns based on similarity. We developed a Python module, “Heatmap.py” (see Supplementary Material) which uses the Python environment within GenGIS to generate a heatmap by using the RPy2 libraries (http://rpy.sourceforge.net) to communicate with R. The resulting heatmap, generated for classes Clostridia, Bacteroidia, and “Unclassified Bacteria” (Fig. 4), shows the rough complementarity between the relative abundance of Bacteroidia and “Unclassified Bacteria” and a clustering similar but not identical to that displayed in Figure 2b. Further custom exploration of data is supported through the GenGIS Python console which allows analyses and visualizations to be implemented with Python scripts or through the R statistical library accessed via RPy2. Although much of the analysis above could be carried out directly in R, GenGIS offers significant advantages by supporting interactive visualizations, performing tree layout in 2D and 3D and by offering an interface that automates many statistical tasks.

### Atlantic Reference Condition Model Plugin

By integrating the GenGIS data model, R modules, and the interactive visualization environment, new plugins can be developed to execute specialized data analysis pipelines. For example, reference condition approach (RCA) models have been developed to support bioassessment for river sites globally. Recently, [22] developed an approach for Atlantic Canada based on the River Invertebrate Prediction and Classification System (RIVPACS) methodology [27], [28]. The regional RIVPACS-based RCA model developed for Atlantic Canada uses biological monitoring data collected from wadeable streams paired with freely available, nationally consistent geospatial data layers with minimal anthropogenic influence. Biological classification of sites was carried out using a discriminant function (DF) approach and prediction of habitat group membership as described in [22]. The model allows the derivation of ecological quality-ratio (O/E) data that describes the richness or diversity of a site relative to its predicted value. We implemented the Atlantic Canada RCA approach as a plugin for GenGIS that includes the trained reference model stored as a cross-platform R file object, which allows use of the plugin with RCA models from other regions. The RCA plugin uses the abundance and taxon names from the sequence data and the location metadata that correspond to the parameters in the RCA model to generate various diversity measures. The O/E ratio for each diversity measure can be visualized as a bar graph for each location on the map, exported as location metadata for use with other GenGIS plugins (e.g. Linear Regression plugin), or output to a text file. Ecological assessment of freshwater ecosystems generally lacks geospatially integrated tools for statistical analysis of biological observations. Integration of the RCA approach in GenGIS is to our knowledge the first application of such a tool within this field and as such, provides a unique opportunity to improve explicit interpretation of such data in a spatial context.

**Figure 5:**
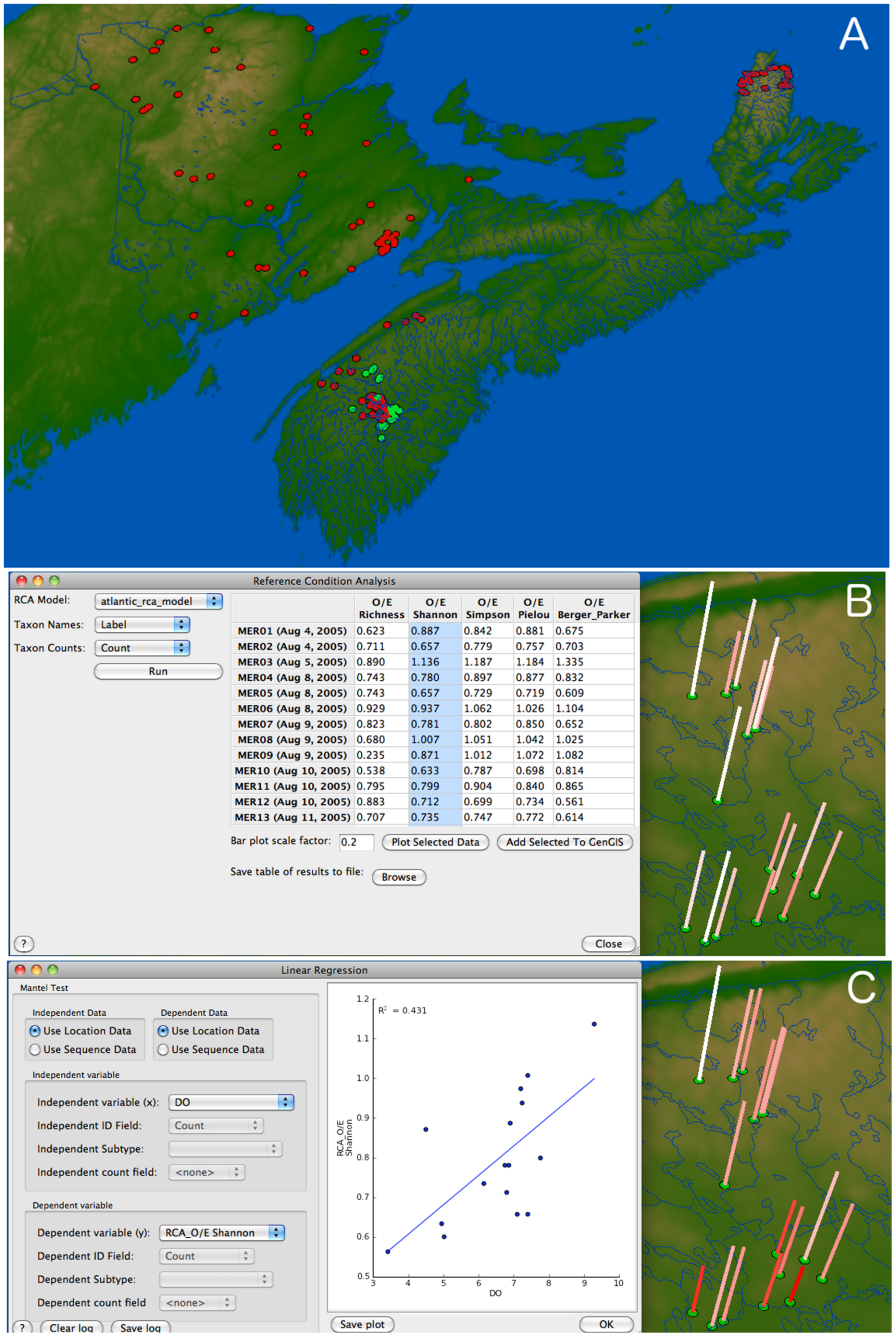
Reference condition analysis. A) The Atlantic RCA model [22] was developed using 128 calibration sites from across the Atlantic Provinces, Canada (red circles) and was run on 16 test sites from the Upper Mersey region, Nova Scotia, Canada (green circles). B) Calculation of the O/E ratio using various diversity measures (table), and O/E for Shannon diversity visualized for each geographic location (bar plots). C) Linear regression of Shannon diversity O/E versus Dissolved Oxygen concentration for each location (r^2^ = 0.431, p = 0.006).

We applied the RCA plugin to 16 test sample sites from the Upper Mersey region in Nova Scotia, Canada (Fig. 5A). The O/E Shannon diversity values calculated by the RCA plugin ranged from 0.563 to 1.136 (Fig. 5B) and were found to correlate with the measured Total Dissolved Oxygen Concentration (mg/L) from these same locations using the Linear Regression plugin (r^2^ = 0.431, p = 0.006). As expected, community diversity was higher at those sites with well-oxygenated conditions, a property of healthy river habitats. By incorporating additional diagnostic tools based on relative abundance, it was possible to observe a stronger relationship between the environmental stressor and the biological endpoint. In fact, when compared to the results obtained by considering only O/E taxa richness, the amount of variance explained nearly doubled (r^2^ = 0.237, p = 0.047; see [22] for details).

### Conclusions

The above examples illustrate many of the new features of GenGIS, including new phylogeographic tests, and an API for plugins that allows users to develop their own tests either as Python/RPy2 scripts that can be executed in the console, or as a new graphical plugin that can easily be used by others. The RCA plugin demonstrates how sophisticated ecological modelling can now be carried out using the R functionality, and how model results can be visualized within a plugin or directly on the geographical display. With the migration of the GenGIS source code to Github, we have made it easier for GenGIS users to make modifications to the software, particularly the development of new plugins, and contribute these changes to the GenGIS codebase. An important future direction of GenGIS is the integration of online data sources to allow the inclusion of reference datasets from, for instance, the Global Biodiversity Information Facility (GBIF: http://www.gbif.org/).

GenGIS lacks the detailed geographic modeling capabilities provided by commercial GIS software such as ArcGIS by ESRI International. Tools such as GeoPhyloBuilder [3], [33] have been developed to provide phylogenetic visualizations within ArcGIS and provide a powerful analytical platform. Other software packages rely on the proprietary Google Earth software for visualizing geographic information such as the spread of diseases [4] or the confidence intervals of Bayesian biogeographical analysis [5]. While these visualizations are often powerful, they are restricted due to their reliance on proprietary software. Online visualizations and analysis tools have also emerged, with the Map of Life [34] exemplifying the use of a shared online database for exploring species distributions. The primary strengths of GenGIS are its ability to quickly integrate multiple data sources, carry out statistical analysis, and perform visualizations to support exploratory analysis and rigorous testing within an easy-to-use, open-source software package. While other tools provide functionality not currently available within GenGIS, the range of analyses and visualizations currently provided by GenGIS will be of value to many researchers and the ability to produce custom plugins allows flexible exploration of data.

### Availability

GenGIS v2 is freely available under the GNU General Public License version 3.0. Executable binaries for Windows and Mac OS X can be obtained at http://kiwi.cs.dal.ca/GenGIS. Source code is hosted at Github (https://github.com/beiko-lab/gengis). The GenGIS website contains an online manual, several written and video tutorials, and links to useful source for digital map data. MapMaker, software which provides maps compatible with GenGIS, can also be obtained on the website.

## Supporting Information

Figure S1 **Degenerate cases for the Linear Axes Analysis algorithm.** a) Multiple samples may be taken from the same geographic locations. b) Sample sites may have the same longitudinal coordinates. c) Multiple pairs of sample sites may have a projection line with the same slope. d) Sample sites may be collinear. In cases b–d, sample sites are laid out along the GLL in the order they would appear after a small clockwise rotation passed the degenerate angle.
(TIF)

Text S1 **Supplementary Material.**
(DOCX)
