# Supplementary figures and images for: GenGIS 2: Geospatial Analysis of Traditional and Genetic Biodiversity, with New Gradient Algorithms and an Extensible Plugin Framework
Source: PLoS One. 2013 Jul 29;8(7):e69885. doi: 10.1371/journal.pone.0069885 (PMC3726740; doi:10.1371/journal.pone.0069885)

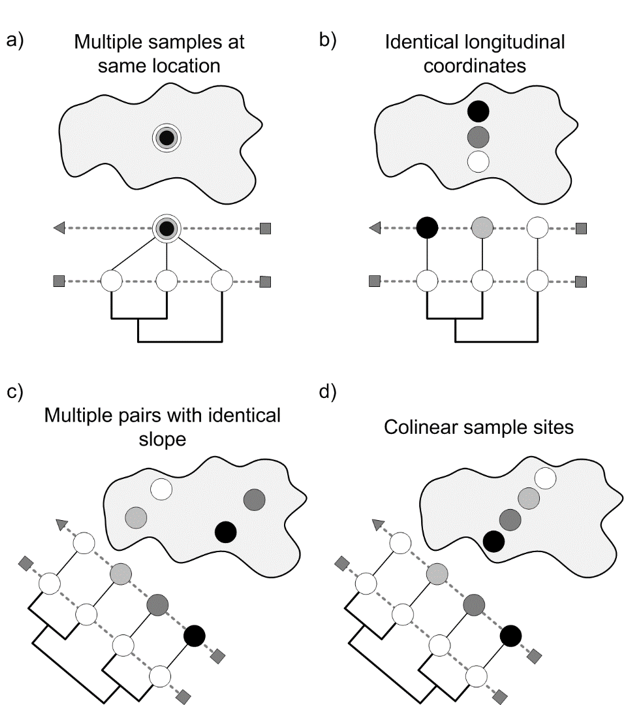

Supplement: Figure S1 — Degenerate cases for the Linear Axes Analysis algorithm. a) Multiple samples may be taken from the same geographic locations. b) Sample sites may have the same longitudinal coordinates. c) Multiple pairs of sample sites may have a projection line with the same slope. d) Sample sites may be collinear. In cases b–d, sample sites are laid out along the GLL in the order they would appear after a small clockwise rotation passed the degenerate angle. (TIF) [file pone.0069885.s001.tif]
