# Supplementary material for: GenGIS 2: Geospatial Analysis of Traditional and Genetic Biodiversity, with New Gradient Algorithms and an Extensible Plugin Framework
Source: PLoS One. 2013 Jul 29;8(7):e69885. doi: 10.1371/journal.pone.0069885 (PMC3726740; doi:10.1371/journal.pone.0069885)
Supplement: Text S1 — Supplementary Material. (DOCX) [file pone.0069885.s002.docx]

**Supplementary Material**

**GenGIS 2: Geospatial analysis of traditional and genetic biodiversity, with new gradient algorithms and an extensible plugin framework**

Linear Axes Analysis Algorithm

**Input**: *sampleSites*, a vector indicating the *x* and *y* position of each sample site; *tree*, a tree where each leaf node is associated with a sample site

**Require**: *calculateSlope*(*rise*, *run*) which calculates a slope between [90°, 270°), *sort* (*vector*, *field*) which sorts elements of *vector* in ascending order of the specified *field*, *minNumberCrossings*(*sampleSites, tree*) which returns the minimum number of crossings for *tree* and a set of sample sites ordered according to the vector *sampleSites*, *swap*(*i*, *j*, *vector*) which swaps the the elements at positions *i* and *j* in *vector*

**Return**: array vector indicating the number of crossings for each permutation of the sample sites

**Notational note**: angles are measured using an azimuth where 90° is due east (i.e., standard compass directions)

**procedure** *LinearAxesAnalysis*(*sampleSites*, *tree*)

{ calculate slope for each pair of sample sites }

*slopeInfoVector* = [ ]

*slopeInfoIndex* = 1

**for** *i* = 1 to |*sampleSites|*

**for** *j* = *i*+1 to *|sampleSites|*

*slopeInfo.slope = calculateSlope*(*sampleSite*[*i*].y – *sampleSite*[*j*].y, *sampleSite*[*i*].x – *sampleSite*[*j*].x)

*slopeInfo.indexI = i*

*slopeInfo.indexJ = j*

*slopeInfoVector*[*slopeInfoIndex*] = *slopeInfo*

*slopeInfoIndex* = *slopeInfoIndex +* 1

**end for**

**end for**

{ sort vector in ascending order of slope }

*sort*(*slopeInfoVector*, *slope*)

{ set initial ordering of sample sites based on their x-coordinate }

*sort*(*sampleSites*, *x*)

*numCrossings* = *minNumberCrossings*(*sampleSites, tree*)

*results* = [ ]

*results*[1].*crossings* = *numCrossings*

*results*[1].*slope* = 90

{ calculate number of crossings for each permutation of sample sites }

**for** *i* = 1 to |*slopeInfoVector|*

*swap*(*slopeInfoVector*[*i*].*indexI*, *slopeInfoVector*[*i*].*indexJ*, *sampleSites*)

*numCrossings* = *minNumberCrossings*(*sampleSites, tree*)

*results*[i+1].*crossings* = *numCrossings*

*results*[i+1].*slope* = *slopeInfoVector*[*i*]*.slope*

**end for**

**return** *results*

Degenerate Cases for Linear Axes Analysis Algorithm

There are 4 degenerate cases which must be considered when implementing the linear axes analysis algorithm. These are not included in the pseudocode above for the sake of conciseness and clarity, but are described below and illustrated in Figure S1.

- *Sample sites at the same geographic location*. Independent samples may be taken at the sample geographic location. Sites with the same geographic location will project to a single point along a linear axis. The *minNumberCrossings* method must be able to handle this case. In our implementation, we detect all such sites at the start of the algorithm and remove all except one from the *sampleSites* vector. Duplicate sample sites are then added back into the *sampleSites* vector just prior to calling *minNumberCrossings*. Layout of the optimal tree accounts for multiple taxa being located at the same sample site. For visualization purposes, each taxon is given a unique node along the user-defined geographic axis. In order to ensure an optimal layout is achieved, all possible orderings of the taxa are considered and the ordering resulting in the fewest crossings is retained. For the majority of data sets this is an acceptable solution, although in extreme cases this may result in unacceptably long running times. This same criterion is applied when constructing the null model for the permutation test.
- *Identical longitudinal coordinates (x-coordinates)*. If multiple sample sites have the same x-coordinate, extra work must be done when setting the initial ordering of sample sites. Sample sites should be placed in the ordering which occurs when the linear axis is rotated a small ε amount in the clockwise direction (i.e., positioned based on their y-coordinate value).
- *Multiple pairs of points with identical slopes.* Multiple pairs of sample sites may result in lines with the same slope. Handling sets of sample sites which are collinear is described below, but care must be taken even for noncollinear sample sites resulting in the same slope. The *swap* function must be called for all *slopeInfoVector* elements with the same *slope* before calling *minNumberCrossings* and storing the results.
- *Collinear sample sites.* The degenerate case of multiple sample sites projecting to the same position along a gradient is only explicitly handled when sample sites have the same geographic location (see above). For collinear sample sites (or any pair of sample sites), we are only interested in the number of crossings which occur from an ε rotation in either direction. Let θ be the angle of an axis resulting in 3 or more sample sites being collinear (i.e., along a line with an angle of θ+90°). For an angle of θ – ε, all sample sites will be in the correct order. At θ + ε, the ordering of any set of collinear points needs to be reversed.

Supplementary Data Files

File S1 – (Kangaroo-Apple.zip) – Data files for kangaroo apple analysis.

- *Australia.tif*: map of Australia and surrounding region.
- *Australia.tfw*: file describing geographic information for *Australia.tif*.
- *Kangaroo.samples.txt*: locations file for the 21 kangaroo apple samples.
- *Kangaroo.tre*: kangaroo apple phylogeny in Newick format.

File S2 (Costello-Body.zip) – Data files used in human body site analysis.

- *Body_locations_All.csv*: location file for all 28 body sites.
- *Body_locations_Fecal_only.csv*: location file for fecal samples, with locations separated by individual, time point, and sampling technique.
- *Costello_sequences_All.csv*: sequence file for all body sites, with samples aggregated by site.
- *Costello_sequences_Fecal.csv*: sequence file for fecal samples, separated by individual, time point, and sampling technique.
- *Costello_UPGMA_Fecal_only.tre*: Newick-formatted tree file showing the clustering relationships among all 24 fecal samples.
- *Heatmap.py*: Python script to compute R heatmap for selected taxonomic groups.
- *Human_body_silhouette_green.tif*: "map" file for body sites.
